# Inference of Genetic Structure and the Process of Population Formation in Nepalese Native Goats Using Uniparental and Genome-Wide Markers

**DOI:** 10.3390/ani16172641

**Published:** 2026-08-23

**Authors:** Ryo Masuko, Yuka Katayama, Yuto Nomura, Fuki Kawaguchi, Shinji Sasazaki, Manoj Kumar Shah, Jiaqi Wu, Takahiro Yonezawa, Hideyuki Mannen

**Affiliations:** 1 Laboratory of Animal Breeding and Genetics, Graduate School of Agricultural Science, Kobe University, Kobe 657-8501, Japan; ryo.masuko26@gmail.com (R.M.);; 2 National Swine Research Program, Nepal Agricultural Research Council, Pakhribas 56800, Nepal; 3 Graduate School of Integrated Sciences for Life, Hiroshima University, Higashi-Hiroshima 734-8551, Japan

**Keywords:** Nepal, genetic structure, goats, Himalayas, mitochondrial DNA, migration route, sex-determining region Y, 50K SNP

## Abstract

Nepal is a small, landlocked country with marked elevational variation from the Terai plains to the Himalayas. Here, four indigenous goat populations (Chyangra, Sinhal, Khari, and Terai) are raised at different elevations. This study aimed to clarify the genetic structure of these populations and how they are formed and propagated across the Himalayan region. We analyzed 136 Nepalese goats using mitochondrial (mt) DNA D-loop and *sex-determining region Y* (*SRY*) 3′-untranslated region (UTR) sequences, as well as 50 K SNP array data. The mtDNA haplogroups D (0.162) and G (0.03) were detected only in Chyangra, whereas haplogroup B was predominant in Sinhal (0.42), followed by Khari (0.260). Regarding *SRY* haplotypes, Y2B was detected in all populations, whereas Y1AB (0.42) was found only in Chyangra. Genome-wide SNP analysis showed that Chyangra was genetically related to Tibetan and Central Asian goats, while Terai resembled South Asian goats. Interestingly, Sinhal formed a distinct cluster, whereas Khari exhibited an admixed genetic structure. These findings suggest that Nepalese goats originate from at least three ancestral lineages and that an additional migration route may have existed through the southern Himalayas.

## Simple Summary

South Asia is believed to be one of the goat migration routes; however, the details of the route remain largely unknown. Nepal has highly diverse environments, ranging from the lowland Terai plains to the high Himalayan mountains, where four indigenous goat populations, Chyangra, Sinhal, Khari, and Terai, are raised separately at different altitudes. In this study, we investigated the genetic backgrounds of these populations using maternal/paternal DNA and genome-wide DNA markers. Our results revealed that each goat breed has a distinct genetic structure. Chyangra was genetically related to Tibetan and Central Asian goats, while Terai was genetically related to Indian goats. In contrast, Sinhal formed a distinct group, whereas Khari appeared to have been formed through admixture among these lineages. A previous study has proposed northern, central, and southern migration routes of goats in Asia. In addition, Tibeto-Burman-speaking peoples residing in the Himalayas have been reported to have spread through Nagaland and Bhutan. In light of this background, this study proposes the possibility of goat migration via the southern Himalayas.

## 1. Introduction

Goats (*Capra hircus*) have been raised for various purposes, such as milk, meat, skin, and fiber. They are also highly adaptable to harsh environments, such as high altitudes. Therefore, goats are one of the most important livestock in the world. Archaeological and molecular biological evidence suggests that goat domestication began approximately 9500–11,500 years ago in the Fertile Crescent. Archaeological evidence suggests that this process involved a gradual transition from hunting wild bezoars to the management of goat herds. Domesticated goats most likely originated from the bezoar (*Capra aegagrus*), their principal wild ancestor, in a broad region extending from eastern Turkey to Pakistan. Following domestication, goats spread across Eurasia through multiple migration routes, and genetic studies have suggested several pathways of dispersal toward Central and East Asia [1,2,3,4,5,6,7].

Nepal is a landlocked country, bordered by India to the east, west, and south, and by the Tibet Autonomous Region of China to the north. This country is marked by its vast north–south elevation difference, ranging from the Terai plains (60 m) to Mount Everest (8848 m). The Himalayan region, including Nepal, is one of the world’s most complex linguistic areas. Nepal has over 100 ethnic groups belonging to the Indo-European and Tibeto-Burman language families [8]. Despite the large Buddhist population due to Tibetan influence, Hinduism is the predominant faith. Consequently, goats are highly valued in Nepal as a livestock species for their meat, milk, and wool.

There are four indigenous goat populations in Nepal, with distinct altitude-based breeding regions: the high-mountain Chyangra, the mountainous Sinhal, the hilly Khari, and the lowland Terai. These goats are mainly raised for meat and milk production and show distinct phenotypic characteristics. In particular, goats raised at higher altitudes generally have longer hair, whereas those in lower-altitude regions have shorter hair. However, the detailed origins and formation histories of these four indigenous populations remain poorly understood. Previous studies using molecular analysis identified gene polymorphisms associated with breeding altitudes [9]. However, studies on goat populations in the Himalayan region mainly analyzed populations in neighboring countries of Nepal [10,11]. Therefore, investigating Nepalese goat populations is crucial for estimating genetic relationships and migration routes across the Himalayan region.

Mitochondrial DNA has long been used to trace maternal lineages in domestic goats, revealing six major haplogroups with distinct geographic distributions [4,12,13]. Y-chromosomal markers have subsequently provided complementary information on paternal lineages and have revealed stronger geographic structuring than mtDNA [14]. Microsatellite markers have also been widely applied to assess genetic diversity and population structure, although they are limited by the number of loci analyzed. More recently, genome-wide SNP arrays have enabled higher-resolution analyses of genetic diversity, population structure, admixture, and dispersal in goats [10,15,16]. Thus, combining mtDNA, Y-chromosomal markers, and genome-wide SNPs provides complementary information on maternal, paternal, and autosomal genetic histories. mtDNA and the *sex-determining region Y* (*SRY*) gene serve as important markers for inferring the maternal and paternal lineages, respectively, because homologous recombination does not occur in these regions. Furthermore, analysis of autosomal SNP variants helps reveal the genetic structure of current populations and infer the degree of gene flow among populations.

Hence, in this study, we analyzed maternal mtDNA, paternal *SRY* gene, and genome-wide SNPs to clarify the following: (1) the genetic structure of four indigenous Nepalese goat populations and (2) the history and process of breed formation through genetic admixture within each breed.

## 2. Materials and Methods

### 2.1. Samples

**Figure 1:**
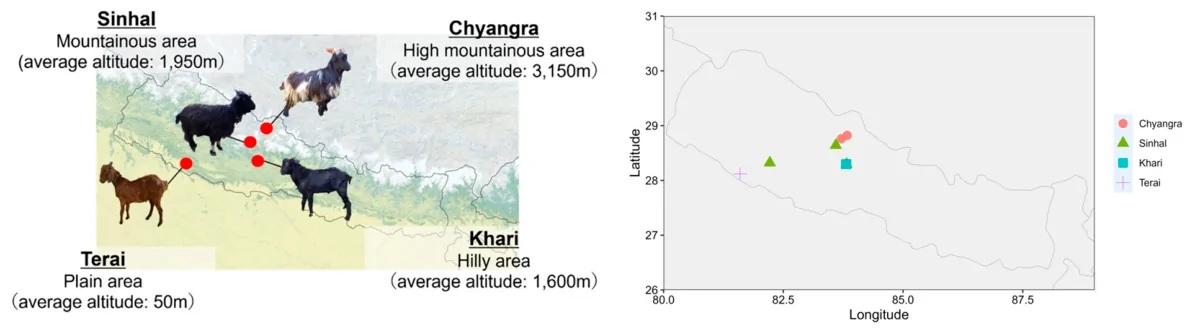
Sampling location and breeding altitude of four Nepalese populations.

We used DNA collected from 136 Nepalese goats (Chyangra: *n* = 37 [males: *n* = 30, females: *n* = 7], Sinhal: *n* = 29 [males: *n* = 9, females: *n* = 20], Khari: *n* = 34 [males: *n* = 22, females: *n* = 12], Terai: *n* = 36 [males: *n* = 26, females: *n* = 10]) (Figure 1 and Table S1). DNA was extracted from the blood samples using the standard phenol/chloroform method [17]. To minimize the inclusion of closely related individuals, farmers were interviewed prior to sampling, and animals with known parent–offspring or full-sibling relationships were excluded.

### 2.2. Sequencing and Genotyping

The mtDNA Hyper Variable region 1 (HV1) and the *SRY* 3′UTR region (543 bp) were amplified and sequenced by the same method as previous studies [14,18,19,20] (Tables S2 and S3). Standard double-stranded DNA sequencing was performed using 20 ng of the amplified product using BigDye^®^ Terminator version 3.1 Cycle Sequencing Kit (Applied Biosystems, Tokyo, Japan). After cycle sequencing, the sequencing products were purified by EDTA/ethanol precipitation. Briefly, 5 μL of 125 mM EDTA and 60 μL of 100% ethanol were added to each reaction, followed by incubation at room temperature for 15 min and centrifugation at 14,000 rpm for 25 min. The pellets were washed with 60 μL of 70% ethanol, air-dried, resuspended in 20 μL of Hi-Di formamide, and denatured at 95 °C for 2 min. Sequencing was performed using an ABI PRISM^®^ 3100 Genetic Analyzer (Applied Biosystems, Tokyo, Japan). Sequence chromatograms were visually inspected in MEGA 7 using both forward and reverse reads [21].

### 2.3. Maternal and Paternal Statistical Analysis

We conducted sequence alignment of the HV1 region according to a previous study (Tables S2 and S3) [22]. The sequence of the mtDNA HV1 region was aligned using the ClustalW program [23] in MEGA 7.0 [21] based on the mtDNA reference sequence (DDBJ accession no. AF533441.1) [24]. Haplotype diversity (Hd) and nucleotide diversity (π) were calculated for each of the four Nepalese populations and for all Nepalese populations using DnaSP v6.12.03 [25]. Sites containing gaps or missing data were excluded from the calculations. The 95% confidence intervals for Hd and π were calculated as the estimate ± 1.96 × standard deviation, using the sampling variance reported by DnaSP.

The *SRY* haplotype data for native Nepalese goats were previously published. The *SRY* haplotype data for native Nepalese goats were previously published. [14]. In this study, we calculated the frequency of paternal haplotypes in four populations (Chyangra, Sinhal, Khari, and Terai) and inferred relationships between Nepal and other regions. To investigate the genetic relationships between Nepalese goats and other regions, we analyzed the geographic distribution of mtDNA haplogroups and *SRY* haplotypes across Eurasia and Africa. We used 6391 and 2019 individuals, which include the previously published data (Tables S4 and S5) for mtDNA and *SRY* analyses, respectively.

### 2.4. Genotyping, Data Construction and Statistical Analysis of SNP Array

We genotyped the 99 Nepalese goats (Chyangra *n* = 24; Sinhal *n* = 24; Khari *n* = 25; Terai *n* = 26) using the Illumina goat SNP 50K Bead chip (53,347 SNPs) (Illumina, Inc. San Diego, CA, USA) (Table S1).

A total of two datasets (Dataset1 and Dataset2) were constructed for use in SNP array analyses (Table S6). Our data were combined with the previously published goat 50K SNP data sets [10,11,26,27,28,29], but without Saanen, Boer, the cosmopolitan Angora, Europe, Africa, the New World, and the Oceanian populations. After merging, quality control was performed using PLINK v1.9. We excluded SNPs with a call rate < 0.98 and maf < 0.01, removing individuals with call rates < 0.98 and LD pruning (plink indep-parwise: window size 50 Kb, step size 5, r2 threshold 0.04). This procedure left 21,678 SNPs with 1318 individuals representing 28 wild or domestic populations from 18 countries for analysis. This dataset was defined as “Dataset1”. For “Dataset2”, populations with more than 40 goats without Nepalese populations were randomly sampled to 40 individuals per population to reduce ascertainment bias. “Dataset2” consisted of 21,678 SNPs with 780 goats/bezoar (Table S6). Principal Component Analysis (PCA), genetic population structure, and admixture analysis used “Dataset2”. We remapped these Datasets to the goat reference sequence ARS1 [30].

To assess the differentiation between populations, pairwise weighted *F*_ST_ distances between populations were calculated with the R package StAMPP [31]. Observed (*H*o) and expected (*H*e) heterozygosity, and inbreeding coefficient (*F*_IS_) were calculated using PLINK v1.9 [32]. Principal Component Analysis (PCA) was also performed in PLINK v1.9, and the results were plotted using R version 4.2.3 [33]. To analyze the genetic population structure, admixture proportions were estimated for K values ranging from 2 to 20 using ADMIXTURE v1.3.0 [34] with Dataset 2. Reduced representation admixture analysis (RRAA) was performed to suppress the inbreeding bias by reducing the representation of inbred populations [11,29]. Because of inbreeding bias, populations with the highest levels of inbreeding are often classified as ancestral clusters. In addition, sampling bias causes overrepresented populations at low K-values to be inferred as ancestral clusters. Therefore, to counteract the effect of inbreeding bias, we limited the proportion of highly inbred populations in our analysis. To achieve this, we randomly selected six individuals from the Cambodia-M population based on normal admixture results, ran the admixture 100 times for such selections, and averaged the percentages of the inferred genomic structure for each individual. The admixture among populations was inferred using TREEMIX 1.13 [35] with m = 1 to 20 migration edges and bezoars as the outgroup.

To infer the genetic affinities between the four Nepalese populations and other populations, we performed outgroup f3 statistics and f4 statistics analyses with the R package ADMIXTOOLS 2 ver. 2.04 [36,37]. Outgroup f3-statistics were calculated in the form f3 (Bezoar; Nepalese population, Asian population), with Bezoar used as the outgroup. For the f4-statistics, selected populations from China, South Asia, and Southeast Asia were used as comparison populations, including Xinjiang, Inner Mongolia, Shandong, Yunnan, Jiangxi, Pakistan, India, Bhutan, Bangladesh, Myanmar, and Laos. The f4-statistics were calculated in the form f4 (Bezoar, pop2; Nepalese population, pop4), where pop2 and pop4 represented either the other Nepalese populations or the selected Asian comparison populations. Statistical significance of the f4-statistics was determined at *p* < 0.001.

## 3. Results

### 3.1. Maternal and Paternal Genetic Analysis of Nepalese Goats

**Figure 2:**
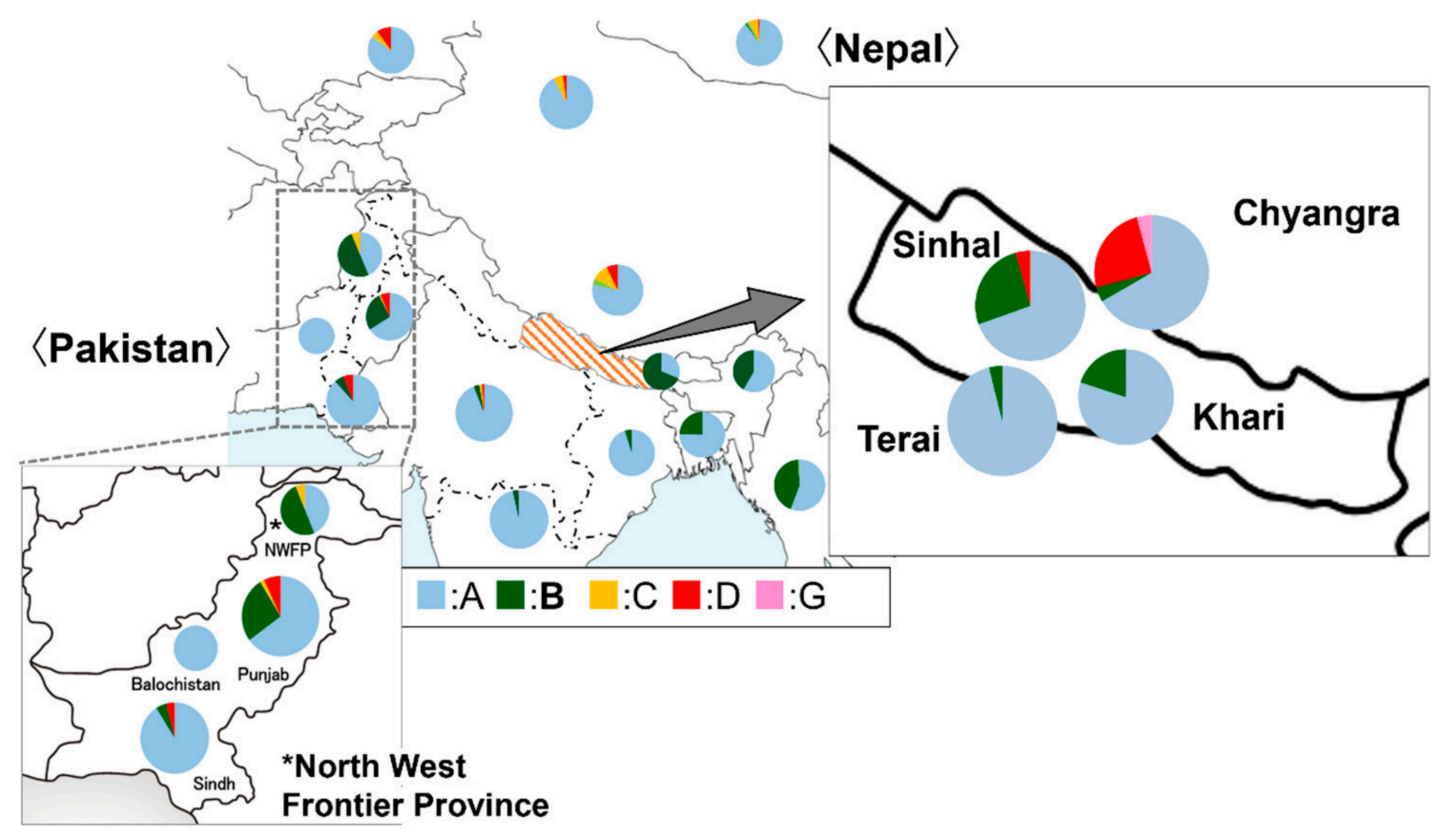
Geographic distribution of caprine mtDNA haplogroups in Nepal and its surrounding region. The size of nodes is proportional to the number of goats present in the node. Colors show the mtDNA haplogroups. The map was generated using Microsoft PowerPoint 2019 based on a free map website by 3kaku-K (https://www.freemap.jp/item/world/world1.html (accessed on 20 August 2026)).

A total of 136 Nepalese goats were sequenced for the mtDNA HV1 region (481 bp). Analysis of these sequences using previously published mtDNA sequences (Accession number AF533441.1) revealed 102 variant sites and 77 mitochondrial haplotypes in Nepalese goats (Figure S1). Furthermore, we constructed a neighbor-joining tree with 22 reference goat sequences [13]. Based on this, 136 Nepalese goats were classified into five haplogroups: A (*n* = 111), B1 (*n* = 16), B2 (*n* = 1), D (*n* = 7), and G (*n* = 1) (Figure 2, Figures S2 and S3, Table 1).

In the mtDNA haplogroup frequencies for four Nepalese populations, Chyangra populations were classified into four haplogroups: A (*n* = 29), B2 (*n* = 1), D (*n* = 6), and G (*n* = 1). Although haplogroup A was predominant, the frequency of haplogroup D was also relatively high. In Sinhal goats, three haplogroups were found: A (*n* = 20), B1 (*n* = 8), and D (*n* = 1). Khari goats were classified into two haplogroups: A (*n* = 27) and B1 (*n* = 7), with frequencies similar to those of the Sinhal population. Terai goats were classified into two haplogroups: A (*n* = 35) and B1 (*n* = 1). Compared with the frequencies of other haplogroups in other Nepalese populations, haplogroup A was remarkably high (Figure 2).

Genetic diversity based on the mtDNA HV1 sequences was high across the Nepalese goats, with an overall haplotype diversity (Hd) of 0.985 (95% CI: 0.979–0.991) and nucleotide diversity (π) of 0.03084 (95% CI: 0.02702–0.03466). At the population level, Hd and π were 0.976 (95% CI: 0.950–1.000) and 0.03144 (95% CI: 0.02482–0.03806) in Chyangra, 0.897 (95% CI: 0.828–0.966) and 0.03536 (95% CI: 0.02874–0.04198) in Sinhal, 0.961 (95% CI: 0.933–0.989) and 0.03207 (95% CI: 0.02522–0.03892) in Khari, and 0.951 (95% CI: 0.918–0.984) and 0.01912 (95% CI: 0.01448–0.02376) in Terai, respectively.

**Figure 3:**
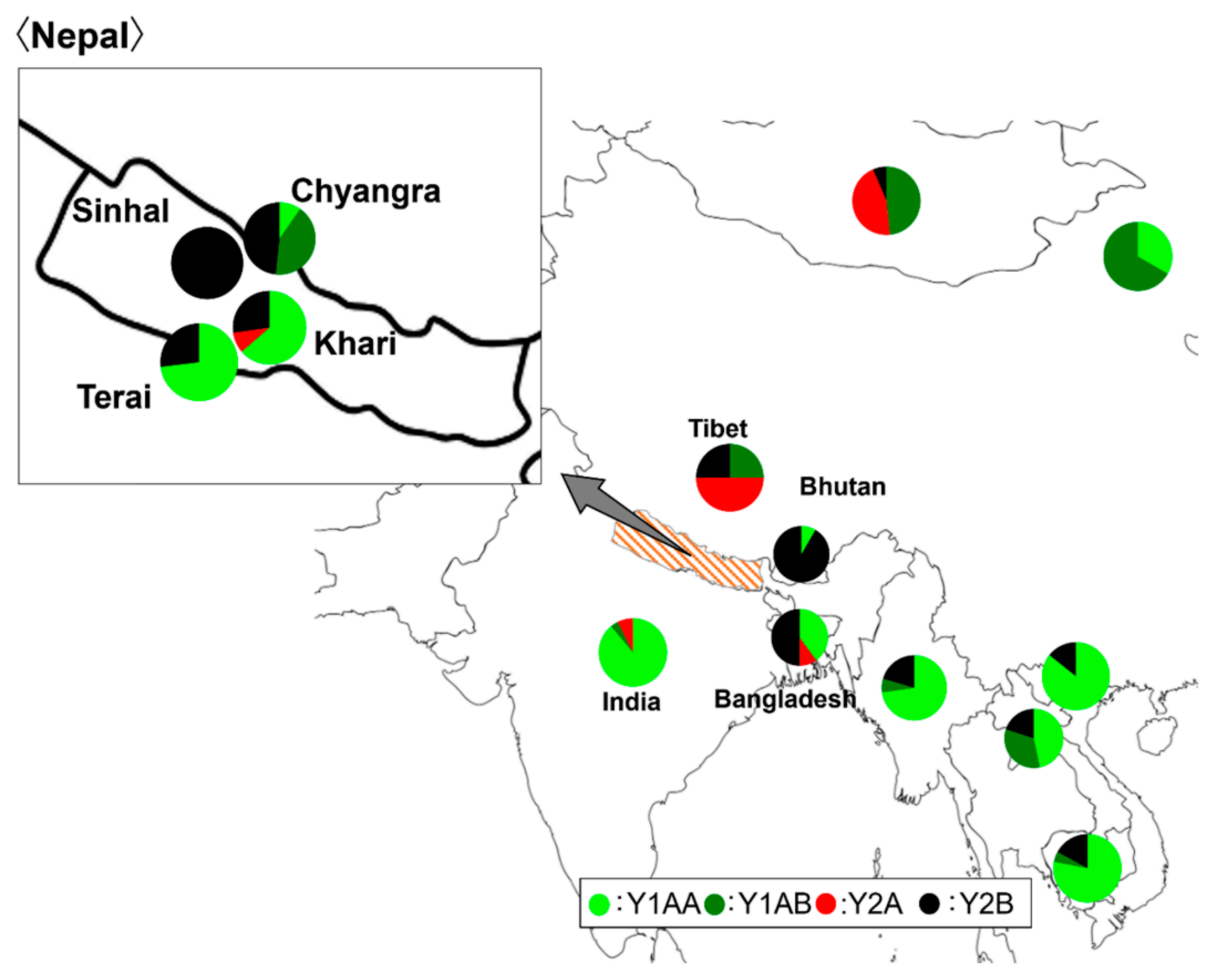
Geographic distribution of caprine *SRY* haplotypes in Nepal and its surrounding region. Colors show the *SRY* haplotypes. The size of nodes is proportional to the number of goats present in the node. The map was generated using Microsoft PowerPoint 2019 based on a free map website by 3kaku-K (https://www.freemap.jp/item/world/world1.html (accessed on 20 August 2026)).

The *SRY* haplotype frequencies for four Nepalese populations are shown in Figure 3, Figure S4 and Table 1. Y1AA (*n* = 36/88), Y1AB (*n* = 13/88), Y2A (*n* = 2/88), and Y2B (*n* =37/88) were observed in Nepalese goats: Chyangra (Y1AA: *n* = 3/31; Y1AB: *n* = 13/31; Y2B: *n* = 15/31); Sinhal (Y2B: *n* = 9/9); Khari (Y1AA: *n* = 14/22, Y2A: *n* = 2/22, Y2B: *n* = 6/22); Terai (Y1AA: *n* = 19/26, Y2B: *n* = 7/26). Y1AA was observed in three populations, except in Sinhal; Y1AB was only in Chyangra (*n* = 13/31); and Y2A was only in Khari (*n* = 2/22). Y2B was observed in all Nepalese populations, with the highest frequency in Sinhal (*n* = 9/9).

### 3.2. 50K SNP Array Analysis

In Asia, the expected heterozygosity (*H*_e_) ranged from 0.196 (Cambodia-M) to 0.444 (Russia), while the observed heterozygosity (*H*_o_) ranged from 0.133 (Cambodia-M) to 0.429 (Turkey) (Table 2). In the four Nepalese populations, *H*_e_ ranged from 0.381 (Sinhal) to 0.414 (Khari), with an overall Nepalese value of 0.402. *H*_o_ ranged from 0.382 (Sinhal) to 0.410 (Terai), with an overall Nepalese value of 0.398. While the three populations, Chyangra, Khari, and Terai, showed similar values, Sinhal exhibited lower values than the other populations (*H*_e_: 0.381; *H*_o_: 0.382). *F*_IS_ ranged from −0.0241 (Kyrgyzstan) to 0.0997 (Laos) (Table 2). In the four Nepalese populations, the *F*_IS_ ranged from −0.0045 (Sinhal) to 0.0255 (Khari).

**Figure 4:**
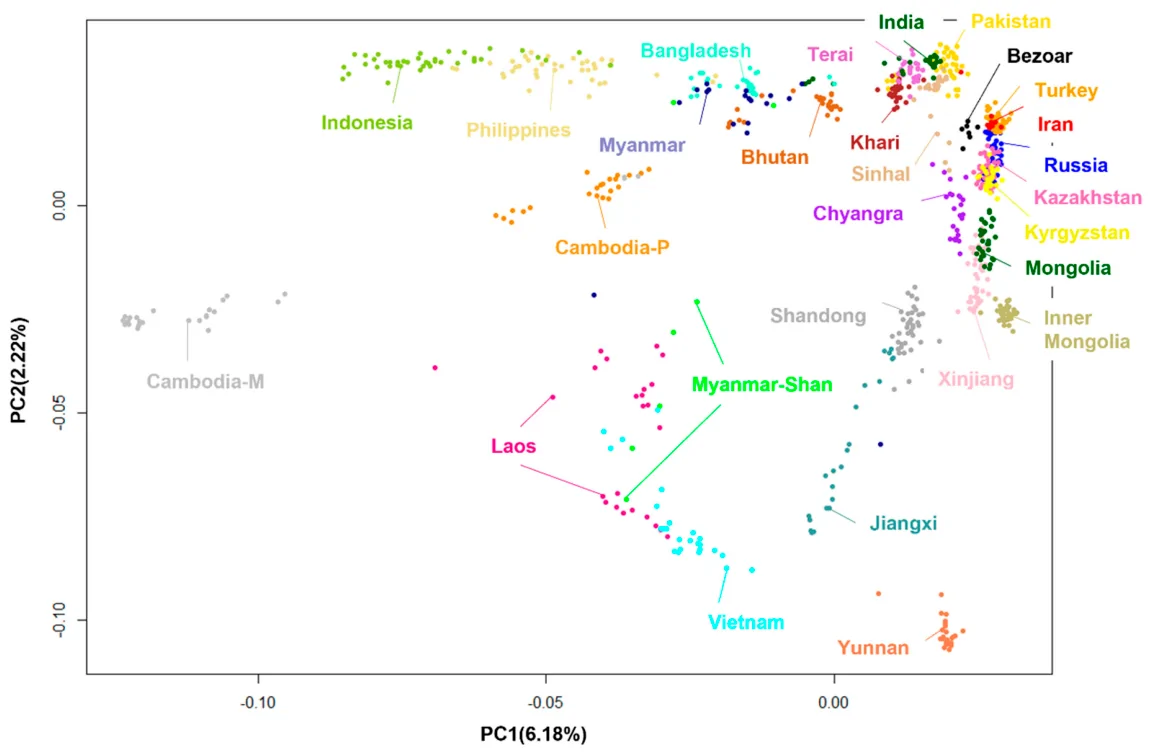
Principal component analysis (PCA) plot of the Asian domestic goats and bezoars. The figure was generated with RStudio 2023.09.0+463 “Desert Sunflower”.

PCA was performed to investigate genetic similarity or genetic structure between and within groups (Figure 4). PC1 and PC2 explained 6.18% and 2.22% of the total genetic variation, respectively, and were selected for visualization because they were the two major components reflecting the geographic relationships among populations. The PCA reflected geographic relationships, with Central Asian and South Asian populations forming distinct clines. Among the four Nepalese populations, Chyangra clustered closer to the Central Asian group, whereas Khari and Terai clustered with the South Asian group. Most Sinhal individuals formed a relatively distinct cluster between these groups, although some overlap among the Nepalese populations was observed.

To estimate the genetic structure within populations and genetic admixture between populations, ADMIXTURE analysis was performed with the number of ancestral populations set to K = 2 to 20 (Figure S5). Cross-validation errors indicated that K = 19 had the lowest error value (0.65016), making it the most likely number of ancestral components. However, as this study primarily aimed to understand the fundamental population processes and genetic structure of Nepalese populations, we focused on interpreting results from models with a small number of ancestral components. At K ≥ 2, a Cambodian-M-derived component (orange) became prominent across South and Southeast Asia, consistent with previous reports [11,29], raising the possibility that the highly inbred Cambodia-M population influenced the ADMIXTURE results.

**Figure 5:**
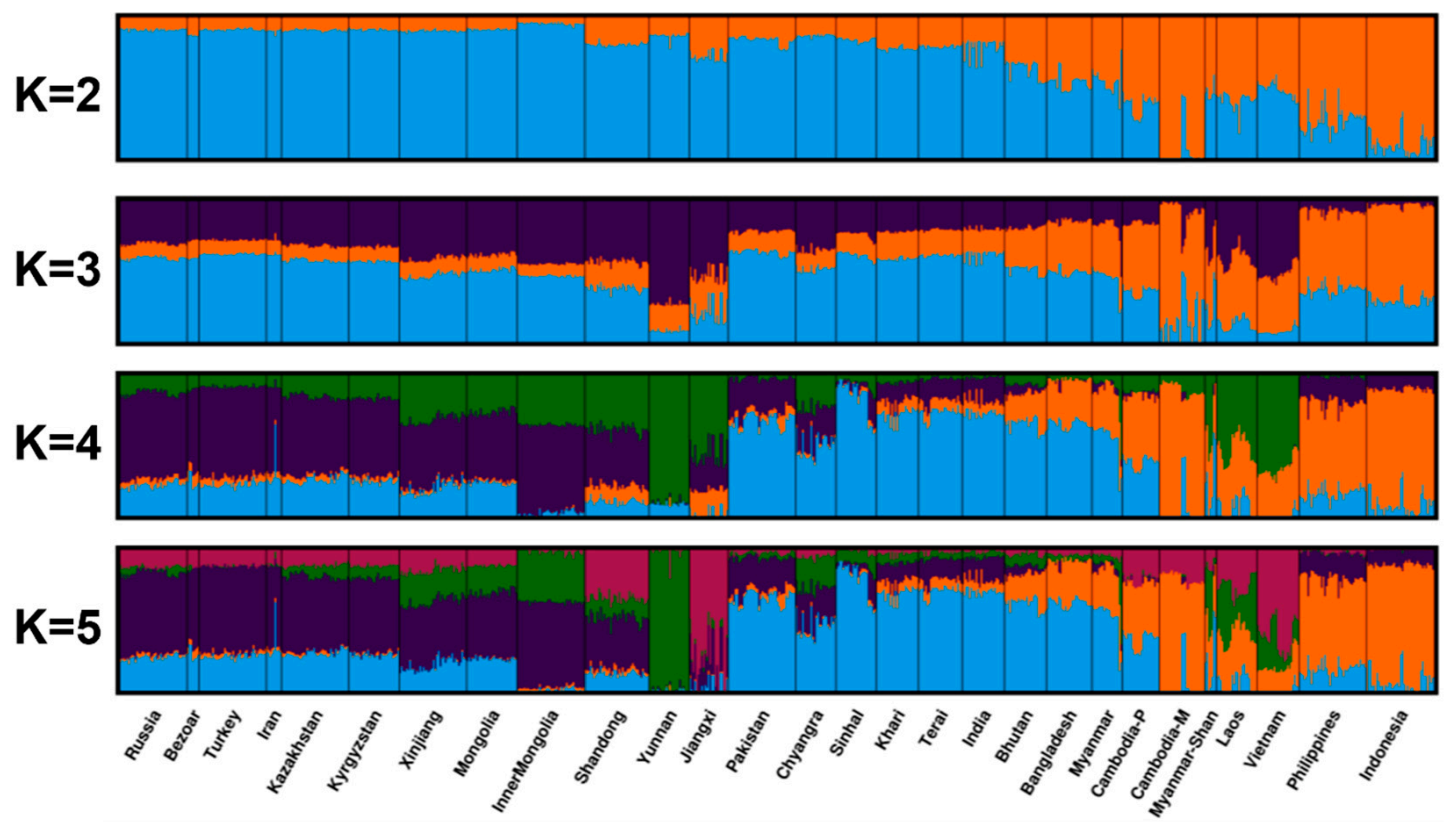
Reduced Representation Admixture analysis (RRAA) of Asian goats at K = 2 to 5 by the ADMIXTURE program ver. 1.3. This figure was generated using CLUMPAK (https://clumpak.tau.ac.il/, accessed on 20 August 2026).

To suppress the inbreeding bias by Cambodia-M, Reduced Representation Admixture analysis (RRAA) was performed (Figure 5 and Figure S6). At K = 2 and 3, RRAA and the normal ADMIXTURE showed identical results. At K ≥ 4, Cambodia-M displayed a pattern similar to that at K = 2 and 3. Regarding the K = 5 result for the four Nepalese populations, all four showed a genetic structure dominated by South Asian genetic components (light blue) (Figure 5). Chyangra showed a higher proportion of genetic components originating from the Middle East and Central Asia (dark blue), Yunnan (Green), and Jiangxi (violet) compared to the other three populations. Sinhal exhibited a higher proportion of the South Asian component and a smaller proportion of other components, suggesting less genetic admixture with other Nepalese populations. Furthermore, Khari and Terai exhibited genetic structures similar to those of the Indian and Pakistani populations.

To assess genetic similarity among populations, the fixation index (*F*_ST_) values were calculated to quantify genetic distances between groups (Table S7). *F*_ST_ values ranged from 0.007 (Turkey/Iran) to 0.410 (Cambodia-M/Bezoar). The mean values for each population ranged from 0.073 (Khari) to 0.286 (Cambodia-M). Among the four Nepalese populations, Sinhal (0.110) had a higher mean value than the other three (Chyangra: 0.087; Khari: 0.073; Terai: 0.079).

To infer population relationships and potential migration events involving the four Nepalese populations, TreeMix was performed with *m* = 0–20 (Figure S7). At *m* = 1 and 2, a topology was plotted using TreeMix to reflect the geographical location. For four Nepalese populations, Chyangra, Khari, and Terai clustered with Central Asian, South/Southeast Asian, and South Asian populations, respectively. Intriguingly, the Sinhal population was plotted between the South Asian population and the South/Southeast Asian cluster. At *m* ≥ 8, a gene flow was inferred between the Chyangra and Yunnan populations. OptM analysis identified m = 1 as the optimal number of migration events.

Outgroup f3-statistics showed that all four Nepalese populations had the weakest genetic affinity with the Turkish population (Chyangra: 0.0346, Sinhal: 0.0348, Khari: 0.0350, and Terai: 0.0354) (Figure S8). In contrast, each Nepalese population showed its strongest genetic affinity with a different neighboring population: Chyangra with Yunnan (0.0487), Sinhal with Bhutan (0.0454), Khari with Bangladesh (0.0478), and Terai with India (0.0487).

To further investigate the genetic relationships of the four Nepalese populations with populations from surrounding regions, f4-statistics were calculated using selected populations from China, South Asia, and Southeast Asia, together with the other Nepalese populations, for comparison (Table S8). Significant asymmetries in allele sharing were detected in comparisons involving Chyangra and Chinese populations, including those from Yunnan and Inner Mongolia [e.g., f4 (Bezoar, Yunnan; Chyangra, Sinhal) = −0.0051, Z = −11.0], and in comparisons involving Khari or Terai and South Asian populations, including those from India and Bhutan [e.g., f4 (Bezoar, India; Terai, Chyangra) = −0.0066, Z = −18.6]. Although the preceding genome-wide analyses did not indicate a strong affinity of Sinhal with any single non-Nepalese population, the f4-statistics detected significant allele-sharing asymmetries involving Sinhal and several South and Southeast Asian populations [e.g., f4 (Bezoar, Pakistan; Sinhal, Chyangra) = −0.0021, Z = −5.71].

## 4. Discussion

Regarding the origins and genetic relationships of the four Nepalese populations, the combined results of maternal (mtDNA), paternal *SRY* lineage, and SNP array analyses revealed that the genetic structures varied among the populations, suggesting different migration routes or genetic influences from different external populations (Figure 2, Figure 3, Figure 4 and Figure 5, Figures S3 and S4, and Table S8). For Chyangra, analysis of mtDNA D-loop sequences revealed that although haplogroup A was common, few individuals belonged to haplogroup D, which was genetically similar to Tibetan populations. Similarly, *SRY* analysis showed that the Y1AB haplotype was observed exclusively in Chyangra. Based on these consistent genetic structures, the Chyangra breed might be genetically related to Tibetan goat populations. Additionally, f-statistics, TreeMix, and RRAA analyses revealed a genetic association between Chyangra and other populations from Yunnan, Central Asia, and the Middle East. These results supported that Chyangra was genetically influenced by Central Asian and northern Chinese populations. This inference can be explained by the geographical proximity of their high-altitude breeding grounds to the Tibetan region and the presence of trans-valley routes, such as those in the Annapurna Conservation Area. Archaeological evidence suggests that humans migrated from Central Asia and Northern China via Tibet into these Himalayan foothills between approximately 3150 and 1250 years ago [38,39]. Furthermore, the presence of historical trade routes such as the Salt Sheep Trail and genetic studies of other livestock, including the Bhyanglung sheep [40], support the inference that Chyangra goats were genetically influenced by Central Asian and Tibetan populations.

The Sinhal and Khari populations exhibited genetic similarities with populations from Northern Pakistan, Bhutan, and Northeastern India. mtDNA analysis revealed that approximately 20% of individuals in these populations belonged to haplogroup B1 (Table 1). The presence of the *SRY* haplotype Y2B in Sinhal suggests a paternal lineage similar to those found in South and Southeast Asia, particularly Bhutan [14]. This genetic pattern is further supported by SNP analysis. In the PCA, Sinhal was positioned between South Asian and South/Southeast Asian populations, consistent with the RRAA results, which showed a genetic structure distinct from the other three Nepalese populations. The f-statistics further suggest that the distinct genetic structure of Sinhal may reflect historical genetic connectivity across the Himalayan region rather than affinity with a single neighboring population. Such broad genetic connectivity is consistent with the possibility that goats were transported along networks extending across the Himalayan foothills. Previous studies have suggested that Tibeto-Burman-speaking peoples spread through Nagaland and Bhutan [41]. Given the broad genetic connections observed around the Himalayan region, Sinhal goats may have been influenced by historical livestock movements along the Himalayan foothills. In contrast to Sinhal, the SNP analysis showed that Khari exhibited genetic continuity and clustering with Terai. Together with the f-statistics and the frequencies of mtDNA and *SRY* lineages, this intermediate genetic structure suggests that Khari may have originated by admixture of both the other three Nepalese populations and neighboring South Asian goat populations.

The Terai population showed a distinct genetic structure closely related to Indian and Bangladeshi populations. Almost all Terai individuals belonged to mtDNA haplogroup A, and the population was composed of Y1AA and Y2B paternal lineages. Moreover, RRAA and f-statistics supported its close genetic relationships with South Asian populations, particularly Indian populations. These genetic similarities can be attributed to the open geography of the Nepal–India border, which allows the free movement of people, goods, and livestock [42]. Additionally, Terai goats share morphological characteristics with Indian populations, such as long, drooping ears. Thus, considering genetic structure, geographical location, and morphological features, Terai goats were inferred to be genetically influenced by Indian goat populations.

Collectively, our study showed that the Sinhal had significantly distinct genetic structures, indicating genetic relationships between Nepal and its surrounding regions, as well as distinct propagation routes. The f-statistics generally corroborated these population-specific patterns, showing stronger genetic affinities of Chyangra with northern Chinese populations, Sinhal with Bhutan, and Terai with India, while Khari showed a more intermediate affinity with South Asian populations. A previous study proposed that the migration routes of Asian goats can be broadly divided into three pathways: North, Central, and South waves (1st with mtDNA-B and 2nd with mtDNA-A) [11]. Given that Chyangra showed genetic similarities to Central Asian and northern Chinese populations, this breed may have spread via the North wave (Kazakhstan–Mongolia–Xinjiang), as previously suggested [11]. Furthermore, Terai, which inhabit lowland regions, showed genetic similarities to Southeast Asian and other South Asian populations, suggesting that this population may have spread via the south wave through India from the Fertile Crescent. Sinhal exhibited an intriguing genetic structure, characterized by a relatively high frequency of mtDNA haplogroup B, the exclusive presence of the *SRY* haplotype Y2B, and a genetic component distinct from those of the other Nepalese populations, as shown using ADMIXTURE analysis. These findings suggest that the South wave, especially the 1st wave, may have passed through Nepal, particularly through the mountainous regions inhabited by the Sinhal. Tibeto-Burman is categorized as a monophyletic group divided from the Sinitic language family and distributed along the southern slopes of the east Alpine–Himalayan orogenic belt [42]. The migration and cultural connections between these language families may have facilitated the propagation of goats from the Western Himalayas to Southeast Asia along the Himalayan belt.

However, several limitations should be considered when interpreting the population history inferred in this study. Although mtDNA, *SRY*, and genome-wide SNPs provide complementary information on maternal, paternal, and autosomal genetic histories, the observed genetic patterns do not necessarily reflect migration routes directly. Historical gene flow and genetic drift may have altered the population genetic patterns over time, thereby masking earlier population relationships. In addition, differences in sampling coverage and the availability of comparative populations may influence the observed patterns. Therefore, the inferred migration and population formation patterns should be interpreted with these limitations in mind. Future studies using whole-genome sequencing and ancient genomic data, together with broader geographic sampling, will provide further insights into the origins, migration routes, and population formation history of Nepalese goats.

## 5. Conclusions

Taken together, our findings suggest that Nepalese goats descended from at least three distinct ancestral lineages. The Chyangra population was likely genetically influenced by the Tibetan population, whereas the Terai population was genetically more similar to the Indian population. The Sinhal population possibly propagated through the Himalayan mountainous regions, corresponding to the 1st South wave. Based on the genetic analyses performed in this study and the distribution of human language families, the 1st South wave of migration likely followed a route through the southern Himalayan regions. In summary, this study provides insights into the propagation and genetic structure of domestic goats in the Himalayas.

## Figures and Tables

**Table 1:** The number of mtDNA haplogroups and *SRY* haplotypes in Nepalese populations.

mtDNA Haplogroups/ *SRY* Haplotypes		Population			
		Chyangra	Sinhal	Khari	Terai
mtDNA					
A		29	20	27	35
B		1	8	7	1
	B1	0	8	7	1
	B2	1	0	0	0
D		6	1	0	0
G		1	0	0	0
*SRY*					
Y1AA		3	0	14	19
Y1AB		13	0	0	0
Y2A		0	0	2	0
Y2B		15	9	6	7

**Table 2:** Observed (*H*o) and Expected (*H*e) heterozygosities, and *F*_IS_ in Asian populations.

Population	*H*o	*H*e	*F*_IS_	Population	*H*o	*H*e	*F*_IS_
Russia	0.428	0.444	0.04	Khari	0.403	0.414	0.03
Turkey	0.429	0.439	0.02	Terai	0.410	0.411	0.00
Iran	0.411	0.418	0.02	India	0.413	0.412	0.00
Kazakhstan	0.428	0.437	0.02	Bhutan	0.362	0.400	0.10
Kyrgyzstan	0.437	0.427	−0.02	Bangladesh	0.397	0.414	0.04
Xinjiang	0.417	0.420	0.01	Myanmar	0.356	0.392	0.09
Mongolia	0.427	0.421	−0.01	Cmbodia-P	0.285	0.344	0.17
InnerMongolia	0.392	0.387	−0.01	Cmbodia-M	0.133	0.196	0.32
Shandong	0.425	0.422	−0.01	Myanmar-Shan	0.315	0.349	0.10
Yunnan	0.336	0.340	0.01	Laos	0.323	0.359	0.10
Jiangxi	0.343	0.364	0.06	Vietnam	0.308	0.345	0.11
Pakistan	0.368	0.421	0.13	Philippines	0.328	0.362	0.09
Chyangra	0.399	0.404	0.01	Indonesia	0.278	0.307	0.09
Sinhal	0.382	0.381	−0.01				

## Data Availability

The data have been deposited in Figshare and DDBJ database. The data are available at Figshare (DOI: https://doi.org/10.6084/m9.figshare.32788350) and DDBJ database (accession numbers: LC856307-LC856442).

## Supplementary Materials

The following supporting information can be downloaded at: https://www.mdpi.com/article/10.3390/ani16172641/s1, Figure S1: mtDNA sequence variation was detected in Nepalese goats. Figure S2: Neighbor-joining tree of Nepalese goats based on 481 bp in the mtDNA HV1 sequence. (●: Chyangra, ●: Sinhal, ●: Khari, ●: Terai, ○: Reference sequence). Figure S3: Geographic distribution of caprine mtDNA haplogroups in the Old World. The size of nodes is proportional to the number of goats present in the node. The used data were shown in Table S4. The map was generated using Microsoft PowerPoint 2019 based on a free map website by 3kaku-K (https://www.freemap.jp/item/world/world1.html (accessed on 20 August 2026)). Figure S4: Geographic distribution of caprine *SRY* haplotypes in the Old World. The size of nodes is proportional to the number of goats present in the node. The used data were shown in Table S5. The map was generated using Microsoft PowerPoint 2019 based on a free map website by 3kaku-K (https://www.freemap.jp/item/world/world1.html (accessed on 20 August 2026)). Figure S5: ADMIXTURE patterns of Asian goats with K = 2 to 20 by the ADMIXTURE program. ver. 1.3. This figure was generated using CLUMPAK (https://clumpak.tau.ac.il/, accessed on 20 August 2026). Figure S6: Reduced Representation Admixture analysis (RRAA) of Asian goats at K = 2 to 10 by the ADMIXTURE program ver. 1.3. This figure was generated using CLUMPAK (https://clumpak.tau.ac.il/, accessed on 20 August 2026). Figure S7: Phylogenetic network of the inferred relationships between Asian populations at m = 1 to 20 using TREEMIX 1.13. Figure S8. Outgroup f3-statistic analysis of Asian goats. We used Bezoar as an outgroup. Table S1: The information of Nepalese goats used in this study. Table S2: Primer information for mtDNA and *SRY* sequences. Table S3: PCR conditions for mtDNA and *SRY* sequences. Table S4: Information of geographic region, country and sample size (N), and number of individuals based on caprine mtDNA haplogroups. Table S5: Information of geographic region, country, sample size (N) and the reference used in analyses of caprine *SRY* haplotypes. Table S6: Sample List used in SNP array analysis. Table S7: Pairwise *F*_ST_ between Asian populations. Table S8: f4 statistics analyses between four Nepalese goat populations and populations from a neighboring region.
