# Supplementary material for: Inference of Genetic Structure and the Process of Population Formation in Nepalese Native Goats Using Uniparental and Genome-Wide Markers
Source: Animals (Basel). 2026 Aug 23;16(17):2641. doi: 10.3390/ani16172641 (PMC13564282; doi:10.3390/ani16172641)
Supplement: Supplementary file 1 [file animals-16-02641-s001.zip › Figure_S.pdf]

	111111111111111111111111111111111111111111111111111111111111111111111111111111111111111111111111111111<br>555555555555555555555555555555555555555555555555555555555555555555555666666666666666666666666666666666<br>777777777777778888888888888888888888888889999999999999999999999999999000000000000000000000000011111111<br>001224566778890001112233344566788888999990011111244446666777777788899000001112222233344444667811244457<br>894165415052401690120223412139224689235798901289945693468234567901219257890280156767912467152238078993	Individuals	Sample No.
AF533441	GCATGATG-GGCTTCTGTACAACAAAGTCTATAGCGCACGAATAAGAGTCTTTATTTATTTCTTCGGATCCTATTGGCAAGGCATTTCAATCATTGGA-CCC		
NP-A01	........-...............G.....G..C.................GC.............................AA...C...........A.A.	8	63,64,65,66,67,68,69,155
NP-A02	........-.....................G..T..................C....GC.........................A.................ATA.	5	119,121,122,123,138
NP-A03	........-.......................GC.......................GC........G...............A...C...........-.A.	4	5,19,30,33
NP-A04	........-...............G.........T.................C....GC.......................A....C...........-.A.	4	6,18,21,35
NP-A05	........-...............G.....G..T.......................CC.......................AA...C...........A.A.	4	80,107,150,151
NP-A06	........-...............G.......GT.................C.....C....T.A......C...T...A...C...........A.A.	4	118,126,137,139
NP-A07	........-.......................C.............A.........GC.........C...................A.........T......A.A.	4	129,130,131,136
NP-A08	..T.....-.......................C.............A.........GC.C...................A...................-.A.	3	4,17,102
NP-A09	........-...........G.T.........C........C......T.......GC........G...............A.................A.A.	3	38,39,45
NP-A10	........-...............G.........T.................C....GC.......A.........A.....A...C.T...........A.A.	3	48,148,149
NP-A11	........-.................A.......T.................C.....C.......................A...C...........A.A.	3	50,53,73
NP-A12	........-...........G.T.........C........C......T.......GC.....T..G...............A.................A.A.	3	52,54,56
NP-A13	........-...........G...........C............A..........GC.......AG...............A...........C....A.A.	3	110,115,133
NP-A14	........-...............G.........C.................C....GC.......................AA...C.......C....A.A.	3	127,128,135
NP-A15	......CA-........C................T.................C....G.........G........A....A..C...............A.A.	2	25,26
NP-A16	........-...............G.........T.....A......A.........GC.......................A...C.......C....A.A.	2	29,34
NP-A17	........-.........................C.....A...........C....GCCT..C.......AA.................A.A.	2	59,62
NP-A18	........-.........................C.....A...........C....GCC......A......C.........AA.................A.A.	2	60,61
NP-A19	........-..........T.G..G.A.......T......................GC.......A.........A.....A...C.T...T......ATA.	2	72,152
NP-A20	........-.........................C.............A.........GC.C...................A.................A.A.	2	104,105
NP-A21	........-.....................G..C.......................G.........C...........A.........T..C...ATA.	2	108,109
NP-A22	........-...........G.............C.......................GC........G...............A.................A.A.	2	111,112
NP-A23	........-.........................C.............A.........GC.....T.A.C...........A.........T......A.A.	2	120,142
NP-A24	...C....-...............G.........T.........G.............G.......................A...C...........A.A.	2	153,154
NP-A25	........-.......................GC.............A........C.C.C...................A.................A.A.	1	1
NP-A26	........-.........................C.............A........CGC.....T...C...........A.........T......-TA.	1	2
NP-A27	........-.........G...............C..T............T.......GC.........C...........A........CT......-.A.	1	3
NP-A28	........-...............G.........C....T........A.T.......GC.......................A...C.T...........-.A.	1	7
NP-A29	........-.........................C.......................GC.........C...........A.........T......ATA.	1	9
NP-A30	........-.....................G..T.......................GC.......A.C...G.........A.........T......A.A.	1	10
NP-A31	........-.........................C.............A.........GC...C.....C...........A.....T...T......ATA.	1	11
NP-A32	........-...............G.........C....T........A.T.......GC.........C...........A...C.T...........A.A.	1	14
NP-A33	........-...............G.........T.............A.........GC..........T......A....A...C...........A.A.	1	23
NP-A34	A.......-..A......................C.............A.........GC.......A.C..CG.........A.........T......A.A.	1	27
NP-A35	........-...............G.........T.............A.........GC.......................A...C.T...........A.A.	1	28
NP-A36	........-.........................C.............A.........GC.C.......C...........A.....T...........A.A.	1	31
NP-A37	A.......-.........................C.............A.........GC.......A.C..CG.........A.........T......A.A.	1	32
NP-A38	A.......-.........................C.............A.........GC.......A.C..CG.........A.........T......A.A.	1	36
NP-A39	........-.........................C.............A.........G..C.....A...............A.................A.A.	1	37
NP-A40	........-...............G.....G..T.......................GC.......................AA...C...........A.A.	1	55
NP-A41	...C....-...............G......A.T...TT..G..G........G...GC.......A........A.....A...C..........A.A.A.	1	58
NP-A42	........-...............G.......GT..................C.....C.......A..........T...A...C...........A.A.	1	74
NP-A43	........-...........G.............C........C.........C....GC........G...............A.................A.A.	1	75
NP-A44	........-.........................C.....A...........C....GCC.............C......AA.................A.A.	1	76
NP-A45	...C....-...............G.........T....T..................GC.......A...............A...C...........A.A.	1	78
NP-A46	........-.........................C........................C.................A.G..A.................A.AT	1	81
NP-A47	........-...............G.........T.................C....GCC.......................AA...C.........A..A.A.	1	101
NP-A48	........-....C..........G.........T.......................GC........G...............A...C...........A.A.	1	103
NP-A49	...C....-...............G.....G..T.........G.............G.......................A...C...........A.A.	1	106
NP-A50	...C....-...............G.........T....T..................GC.......A...............A...C...........A.A.	1	113
NP-A51	...C....-...............G.........T....T..................GC.......A...............A...C...........A.A.	1	114
NP-A52	........-...C................C...C.............A.........GC.......A...............A...........C....A.A.	1	116
NP-A53	........-.A.......................C.............A.........GC.........C...........A.................A.A.	1	117
NP-A54	........-...............G.....G..T....................C..GC.......................AA...C...........A.A.	1	124
NP-A55	........-..............G..C.......T.......................GC..................A...A...C...........A.A.	1	125
NP-A56	........-...............G.........C.......................GC.......A...............A.................A.A.	1	132
NP-A57	........-.........................T.A....G.................GC..................A...A...C...........A.A.	1	134
NP-A58	........-...............G.........C.......................GC.......A...............A...............GA.A.	1	140
NP-A59	...C....-...............G.........T.........G..A..........GC.......................A...C...........A.A.	1	143
NP-A60	........-.........................C........G...A...........C.........C...........A.........T......A.A.	1	145
NP-A61	........-...............G.....G..T.......................GC.......................AA...C...........A.A.	1	146
NP-B101	.T..A...-......C..........A..C...C..T.T......A.A...CC.C.CG.CCT..TA.G..T..C..A...AAT.C..T.....C....A.A.	5	40,41,42,43,44
NP-B102	....A...G......C..........A..C...C..T.T......A.A...CC.C.CG.CCT..TA.G..T..C..A...AAT.C..T.....C....A.A.	2	46,71
NP-B103	....A...-......C..........A..C...C..T.T......A.A...CC.C.CG.CCT..TA.G..T..C..A...AA..C..T.....C....A.A.	2	47,49
NP-B104	....A...-......C..........A..C...C..T.T......A.A...CC.C.CG.CCT..TA.G..T..C..A...AA..C..T.....C....A.A.	2	51,141
NP-B105	....AG..-......C..........A......C..T........A.A...CC.C.CG.CCT..TAAG..T..CC.A...AA..C..T.....C....A.A.	2	77,79
NP-B106	....A...-......C..........A..C...C..T.T......A.A...CC.C.CG.CCT..TA.G..T..C..A...AAT.C..TG....C....A.AT	1	70
NP-B107	....A...-......C..........A..C...C..T.T......A.A...CC.C.CG.CCT..TA.G..T..C..A...AA..C..T.....C....A.A.	1	147
NP-B108	....A...-......C..........A..C...C..T.T......A.A...CC.C.CG.CCT..TA.G..T..C..A...AAT.C..T.....C....A.A.	1	156
NP-B201	....A...-......C..........A.T....C..T........A.A......CCC..CCT..TAA...T..CC.A...AAT.C..T.....C....A.A.	1	22
NP-D01	........-A.....CA..........C..G..CT........G.A.A........C.C...C.T.A...T.....A..GAA.GC..T..C....A..A.A.	2	20,24
NP-D02	........-A.....CA.........A......CT........G.A.A........C.C...C.T.A...T..C..A...AATG...T.GC....A..-.A.	1	8
NP-D03	........-A.....CA.............G..CT........G.A..........C.C...CC..A...T..C..A...AA..G.CT.GC....A..A.A.	1	12
NP-D04	........-A.....C..............G..C.........G.A.A........C.C...C...A...T..C..A...AA..G..T.GC....A..A.A.	1	13
NP-D05	........-A....TCA..........C..G..CT...T....G.A.A........C.C...C.T.A...T..C..A...AATG...T.GC....A..A.A.	1	16
NP-D06	........-A.....CA.............G..CT........G.AGA........C.C...C...A...T..C..A...AA.GC..T.GC....A..A.A.	1	57
NP-G01	........-......C...T.....G.......C..T........A.A..-CC..C...C....T.A.C.T.........A..........GC....A.A.	1	15

Figure S1. mtDNA sequence variation was detected in Nepalese goats.

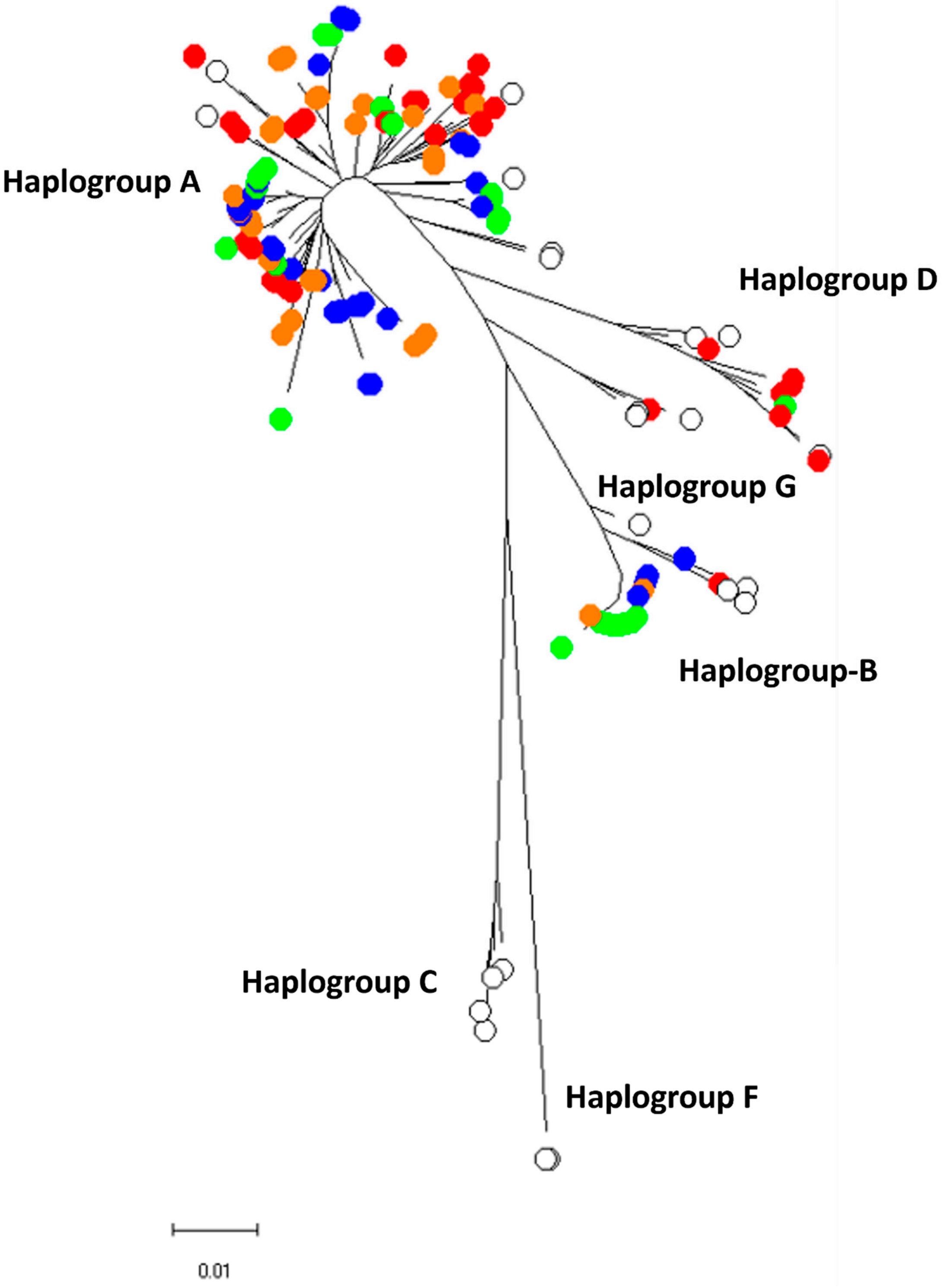

Figure S2. Neighbor-joining tree of Nepalese goats based on 481 bp in the mtDNA HV1 sequence. (●: Chyangra、●: Sinhal、●: Khari、●: Terai、○: Reference sequence)

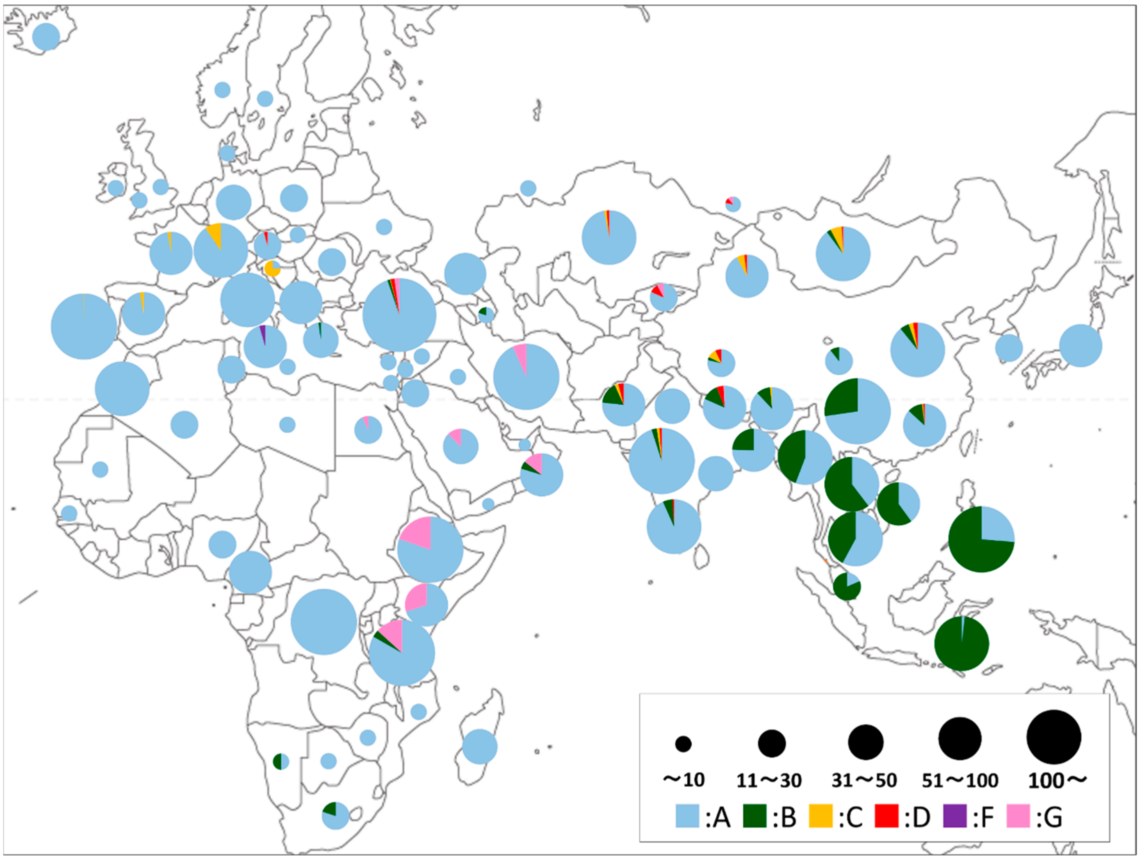

Figure S3. Geographic distribution of caprine mtDNA haplogroups in the Old World. The size of nodes is proportional to the number of goats present in the node. The used data were shown in Table S4. The map was generated using Microsoft PowerPoint 2019 based on a free map website by 3kaku-K (https://www.freemap.jp/item/world/world1.html).

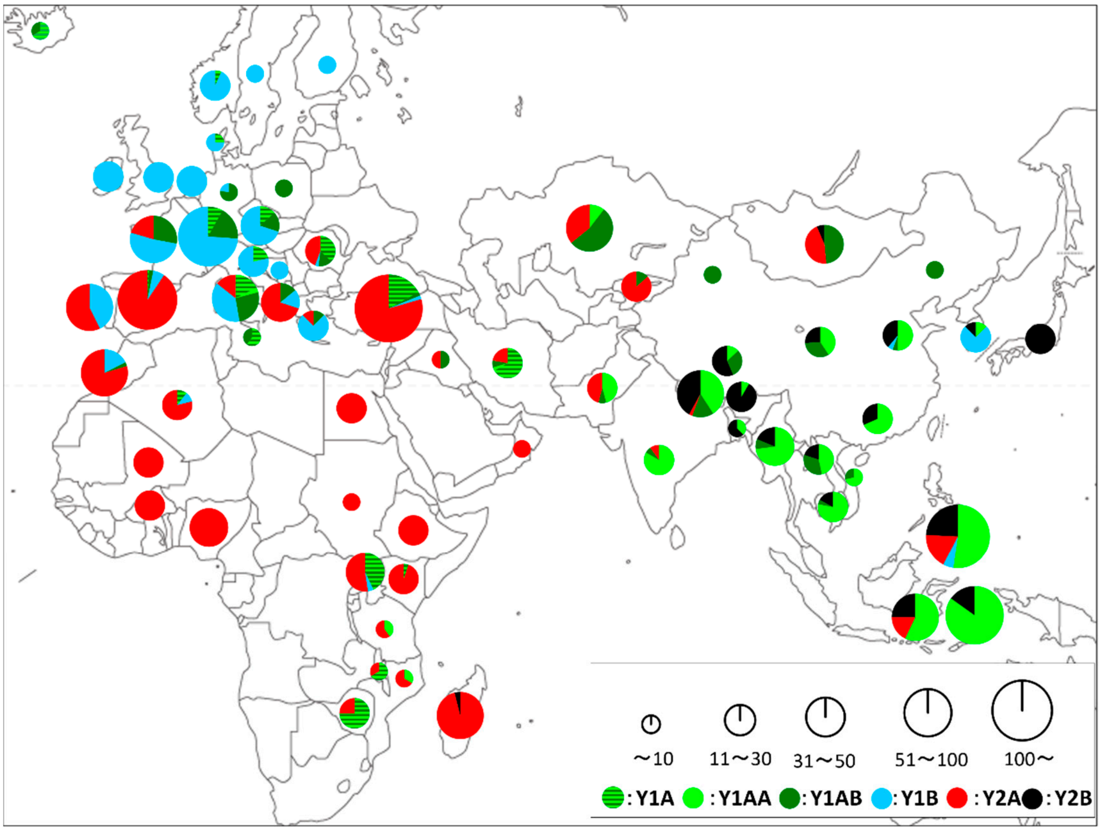

Figure S4. Geographic distribution of caprine SRY haplotypes in the Old World. The size of nodes is proportional to the number of goats present in the node. The used data were shown in Table S5. The map was generated using Microsoft PowerPoint 2019 based on a free map website by 3kaku-K (https://www.freemap.jp/item/world/world1.html).

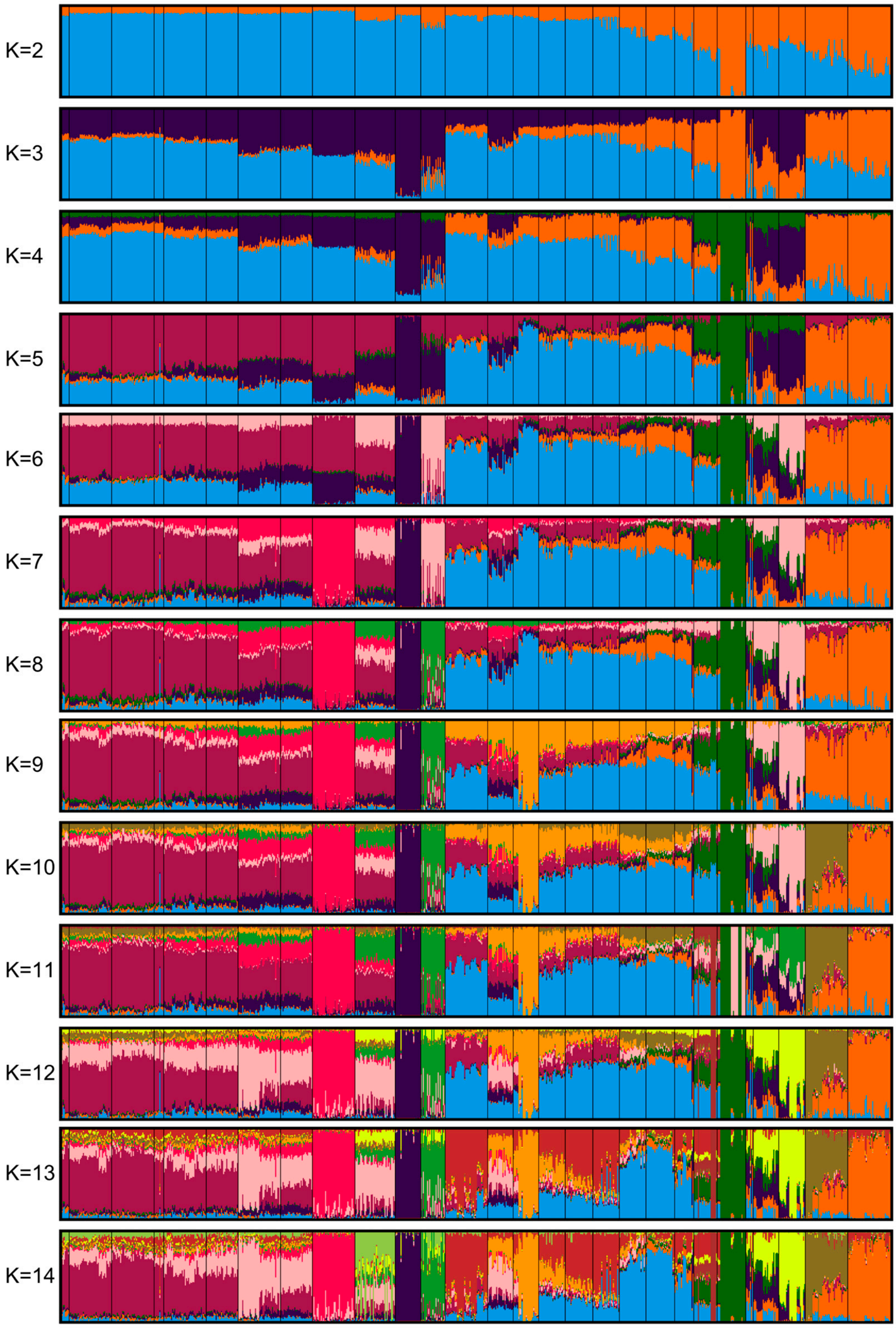

Figure S5. ADMIXTURE patterns of Asian goats with K = 2 to 20 by the ADMIXTURE program. ver. 1.3. This figure was generated using CLUMPAK (https://clumpak.tau.ac.il/).

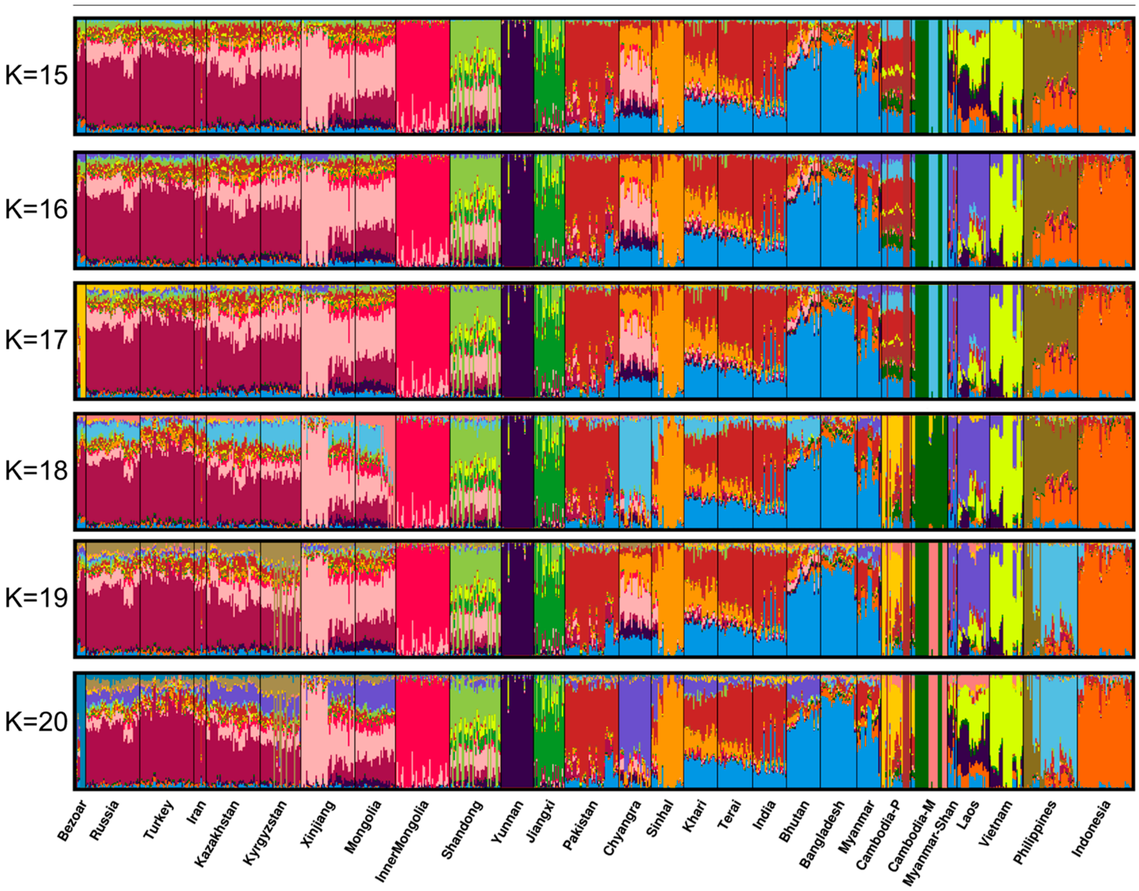

K=15
K=16
K=17
K=18
K=19
K=20
Bezoar
Russia
Turkey
Iran
Kazakhstan
Kyrgyzstan
Xinjiang
Mongolia
InnerMongolia
Shandong
Yunnan
Jiangxi
Pakistan
Chyangra
Sinhal
Khari
Terai
India
Bhutan
Bangladesh
Myanmar
Cambodia-P
Cambodia-M
Myanmar-Shan
Laos
Vietnam
Philippines
Indonesia

(continued).

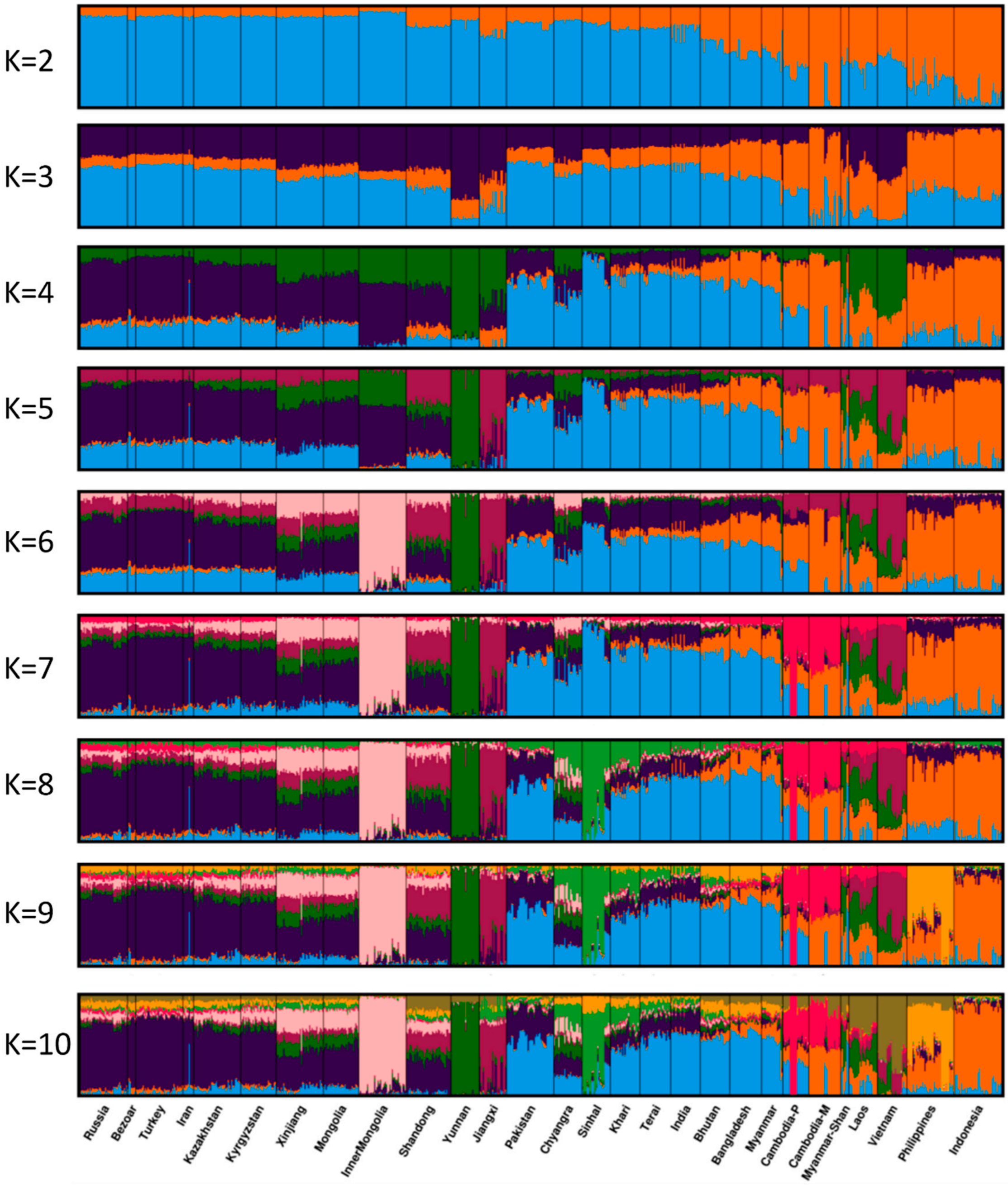

Figure S6. Reduced Representation Admixture analysis (RRAA) of Asian goats at K = 2 to 10 by the ADMIXTURE program ver. 1.3. This figure was generated using CLUMPAK (https://clumpak.tau.ac.il/).

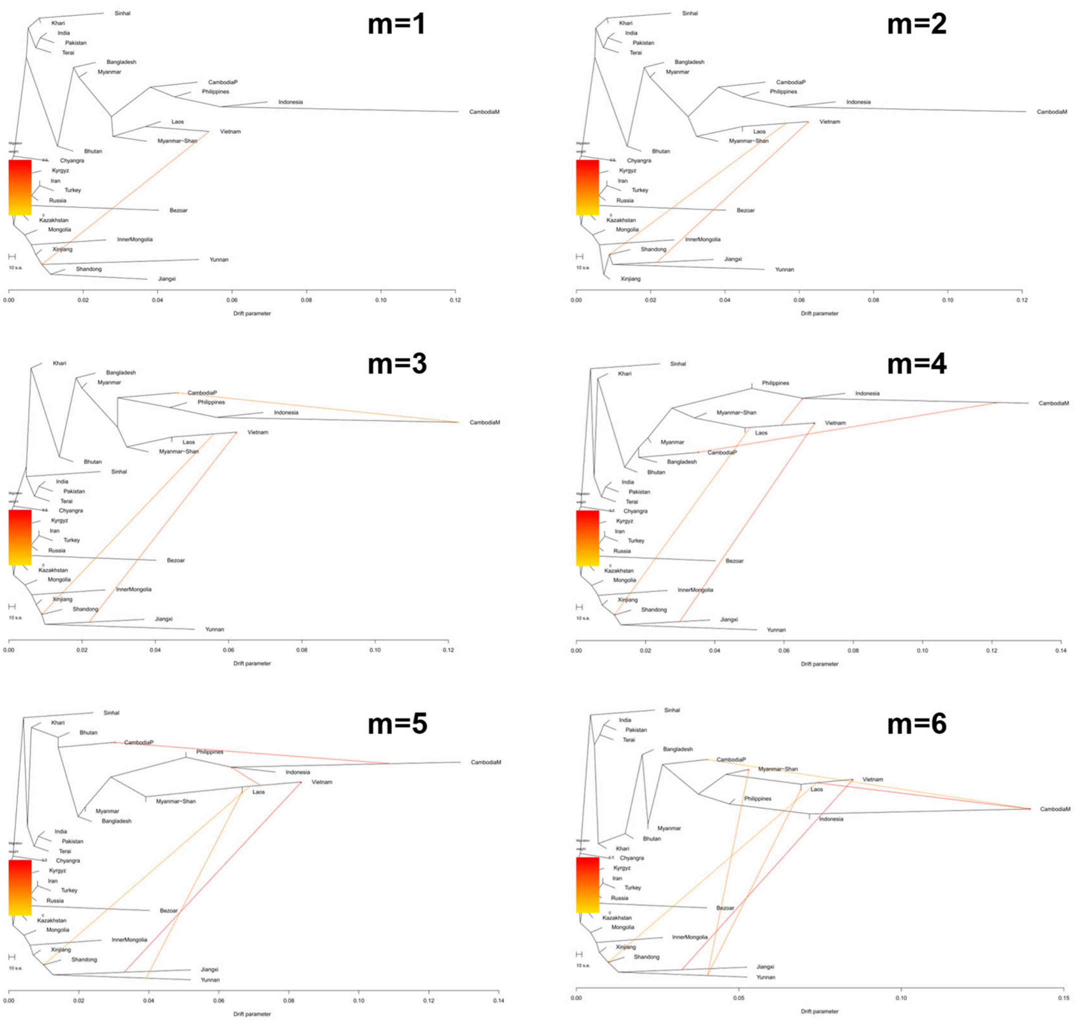

Figure S7. Phylogenetic network of the inferred relationships between Asian populations at m = 1 to 20 using TREEMIX 1.13.

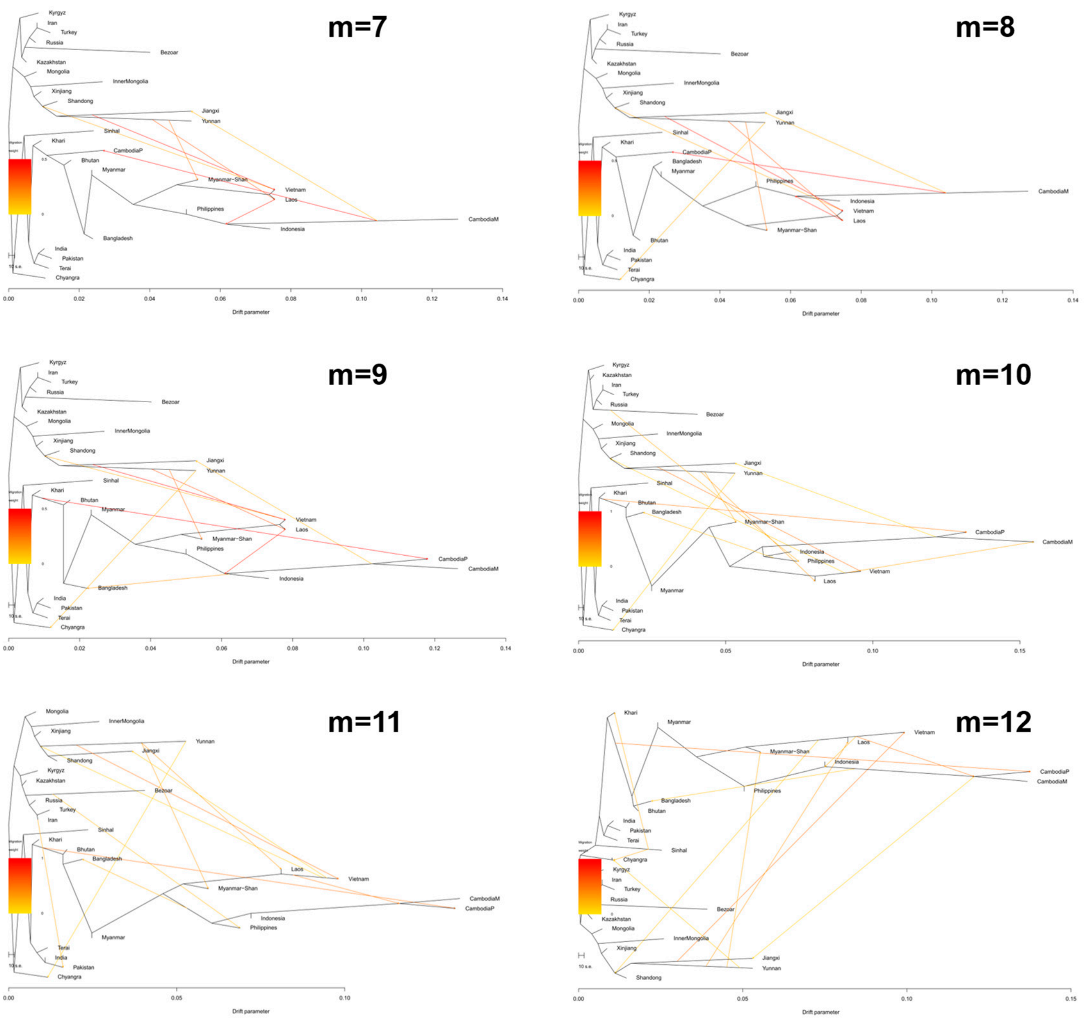

(continued).

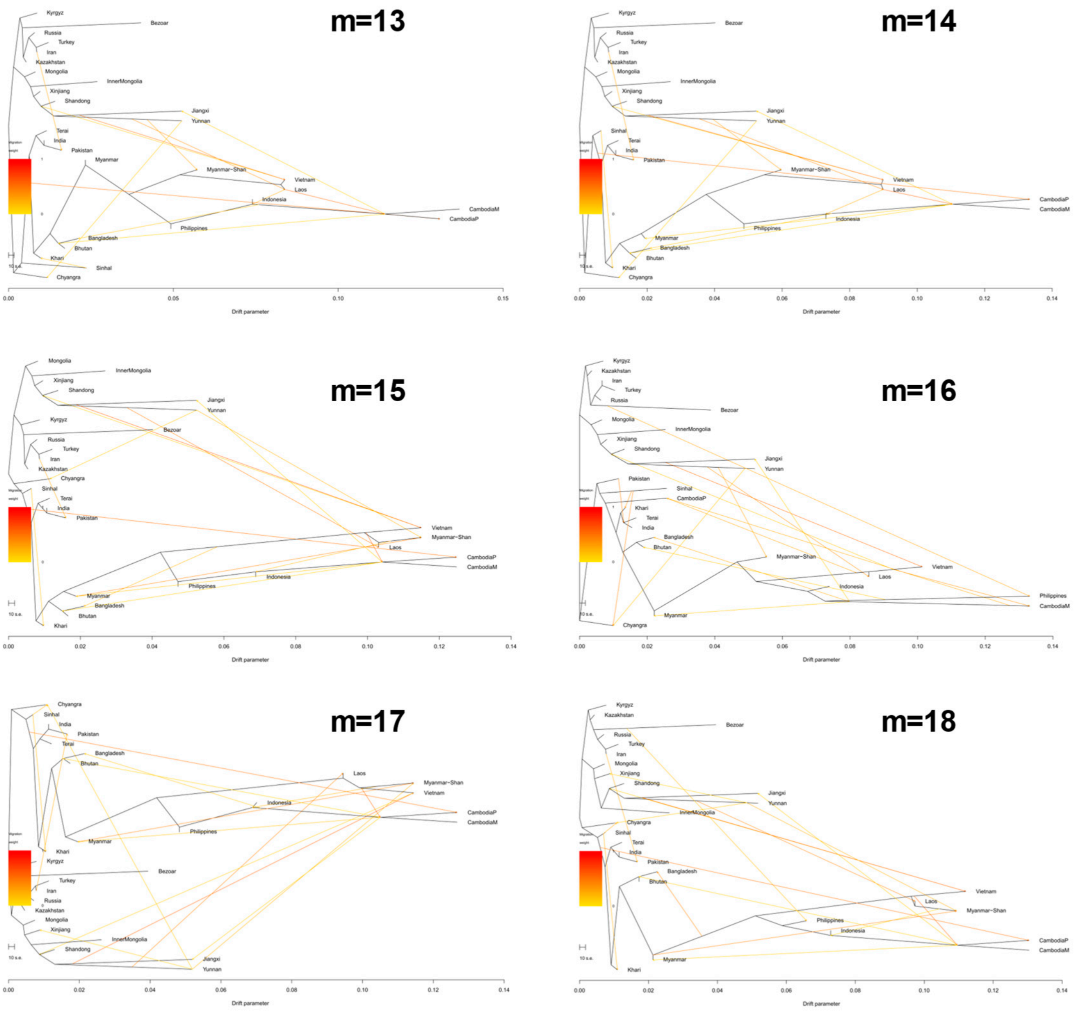

(continued).

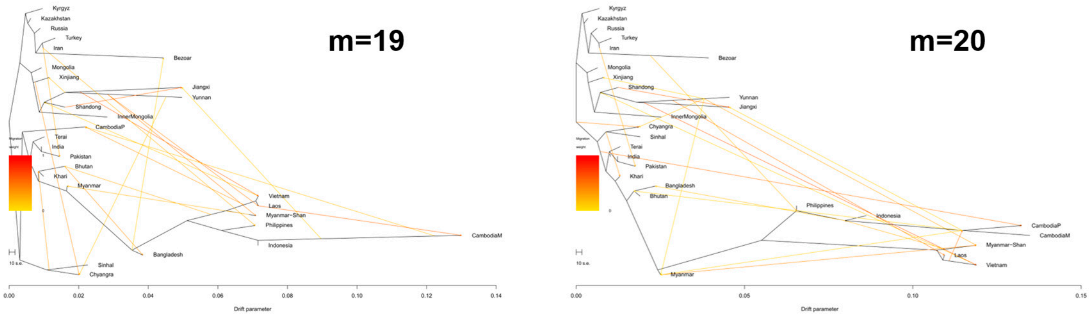
m=19
Kyrgyz
Kazakhstan
Russia
Turkey
Iran
Bezoar
Mongolia
Xinjiang
Jiangxi
Yunnan
Shandong
InnerMongolia
CambodiaP
Terai
India
Pakistan
Bhutan
Khari
Myanmar
Vietnam
Laos
Myanmar−Shan
Philippines
Indonesia
CambodiaM
Bangladesh
Sinhal
Chyangra
10 s.e.
0.00
0.02
0.04
0.06
0.08
0.10
0.12
0.14
Drift parameter
m=20
Kyrgyz
Kazakhstan
Russia
Turkey
Iran
Bezoar
Mongolia
Xinjiang
Shandong
Yunnan
Jiangxi
InnerMongolia
Chyangra
Sinhal
Terai
India
Pakistan
Khari
Bangladesh
Bhutan
Philippines
Indonesia
CambodiaP
CambodiaM
Myanmar−Shan
Laos
Vietnam
Myanmar
10 s.e.
0.00
0.05
0.10
0.15
Drift parameter

(continued).

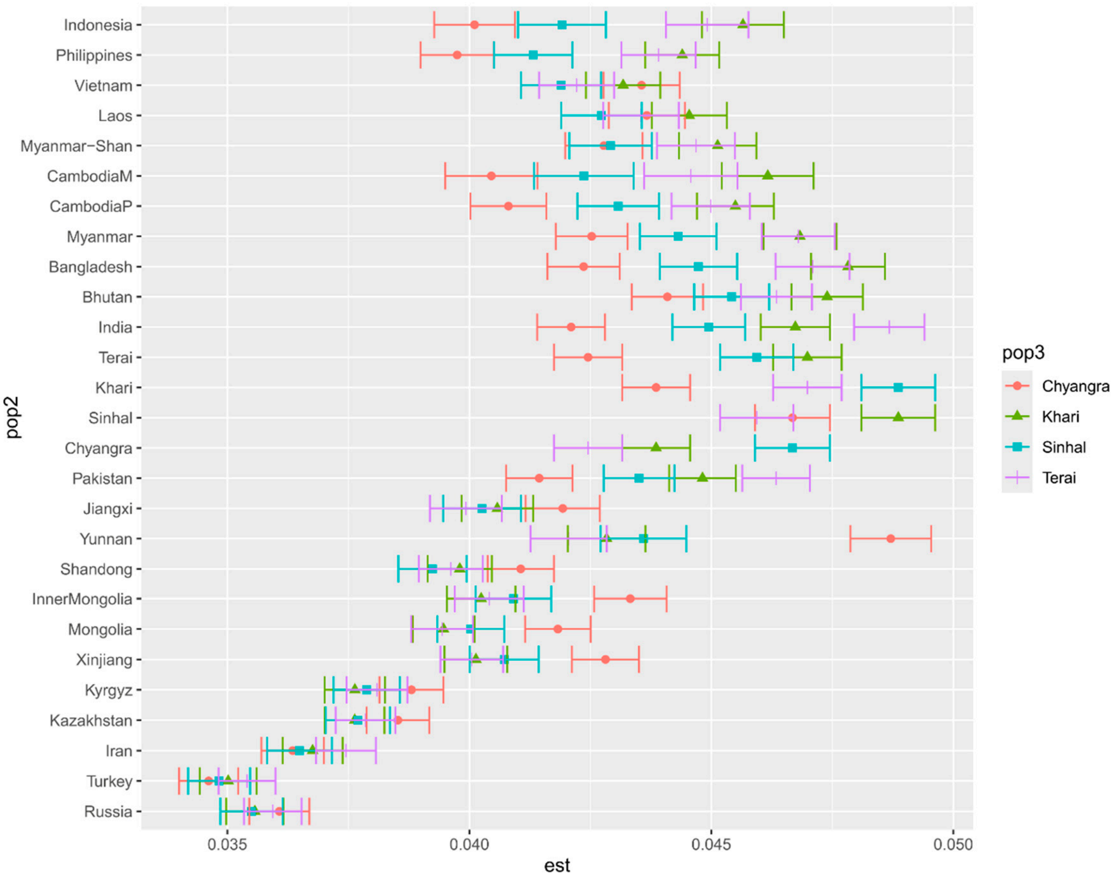

Figure S8. Outgroup f3-statistic analysis of Asian goats. We used Bezoar as an outgroup.
